# LIMK1 and LIMK2 regulate cortical development through affecting neural progenitor cell proliferation and migration

**DOI:** 10.1186/s13041-019-0487-7

**Published:** 2019-07-18

**Authors:** Rui Mao, Rui Deng, Yan Wei, Lifang Han, Yanghong Meng, Wei Xie, Zhengping Jia

**Affiliations:** 1 The Key Laboratory of Developmental Genes and Human Disease, Ministry of Education, Institute of Life Sciences, Southeast University, 2 Sipailou Road, Nanjing, 210096 China; 2 Neurosciences & Mental Health, The Hospital for Sick Children, 555 University Ave, Toronto, ON M5G 1X8 Canada; 3 Department of Physiology, Faculty of Medicine, University of Toronto, 1 King’s College Circle, Toronto, ON M5S 1A8 Canada

**Keywords:** Cortical development, LIMK1, LIMK2, Neuronal proliferation, Migration, Actin

## Abstract

LIMK1 and LIMK2 are key downstream targets to mediate the effects of the Rho family small GTPases and p21-activated kinases (PAK) in the regulation of the actin cytoskeleton. LIMKs are also critical for synaptic transmission, plasticity and memory formation. Changes in LIMK signaling are associated with several neurodevelopmental and neurodegenerative diseases, including autism, intellectual disability and Alzheimer’s disease. However, the role of LIMK signaling in brain development remains unknown. In this study, we used LIMK1 KO and LIMK2 KO mice to investigate the role of LIMK signaling in the cerebral cortical development. We found that these KO mice are reduced in the number of pyramidal neurons in upper cortical layers and this reduction is accompanied by a smaller pool of neural progenitor cells and impaired neuronal migration. These results are similar to those found in PAK1 KO mice and suggest that LIMK-dependent actin regulation may play a key role in mediating the effects of PAK1 and Rho signaling in the regulation of cortical development.

## Introduction

Mammalian neocortex is a major brain area that is critical for various brain functions, including cognition and sensory perception [1–3]. Thus, the precise assembly of neocortex is an essential component of brain development. Abnormities in generation, differentiation or migration of neurons, key processes involved in neocortical development, can cause various neurological and mental disorders, including microcephaly, autism and intellectual disability [4–6]. Although many molecules and signaling pathways such as Reelin and Notch have been reported to be involved, the exact mechanisms underlying precise cortical development remain unclear [7–10]. The actin cytoskeleton is a major structural component of the cell and is required for cell morphology and migration, but its role in mouse brain development is poorly understood [11]. We have previously shown that p21-activated kinase 1 (PAK1), a key target of the Rho family small GTPases essential for actin reorganization, regulates neocortical development by promoting the proliferation of neural progenitor cells and neuronal migration in the cortex [12]. But because PAK1 can target multiple processes in addition to the actin cytoskeleton [13–15], whether the effect of PAK1 on cortical development is mediated by actin remains unknown.

LIM-domain containing kinase 1 and 2 (LIMK1 and LIMK2) are a family of serine/threonine protein kinases that are critical for actin regulation [16–18]. Both LIMK1 and LIMK2 can directly phosphorylate and inactivate the actin depolymerization factor (ADF)/cofilin and promote assembly of actin filaments (F-actin) [19, 20]. This ADF/cofilin-dependent process represents a key mechanism by which the Rho family small GTPase and PAKs regulate actin reorganization [21]. Using knockout mice, we have previously shown that both LIMK1 and LIMK2 are involved in neuronal growth and morphology, synaptic function, pain response and learning and memory [22–26]. However, exactly how LIMK1/2 regulate these processes remain unclear. Because LIMK1/2 are expressed in developing brain [17, 27, 28], we hypothesize that LIMK1/2 may play a role in the development of the brain. In this study, we investigated the role of LIMK1/2 in mouse neocortical development using LIMK1 KO, LIMK2 KO and LIMK1/2 double KO (DKO) mice. We show that these KO mice are significantly altered in neural proliferation, differentiation and neuronal migration in developing neocortex compared to wild type (WT) littermates. These alterations are similar to those observed in PAK1 KO mice [12]. These results provide strong evidence that LIMK/cofilin dependent actin regulation is a major factor in neocortical development. Because LIMK1 is genetically linked to a number of neurological and mental disorders [18], the present study suggests that LIMK1-dependent abnormalities in brain development may contribute to these disorders.

## Results

### Reduced cortical neurons in LIMK KO mice

**Fig. 1:**
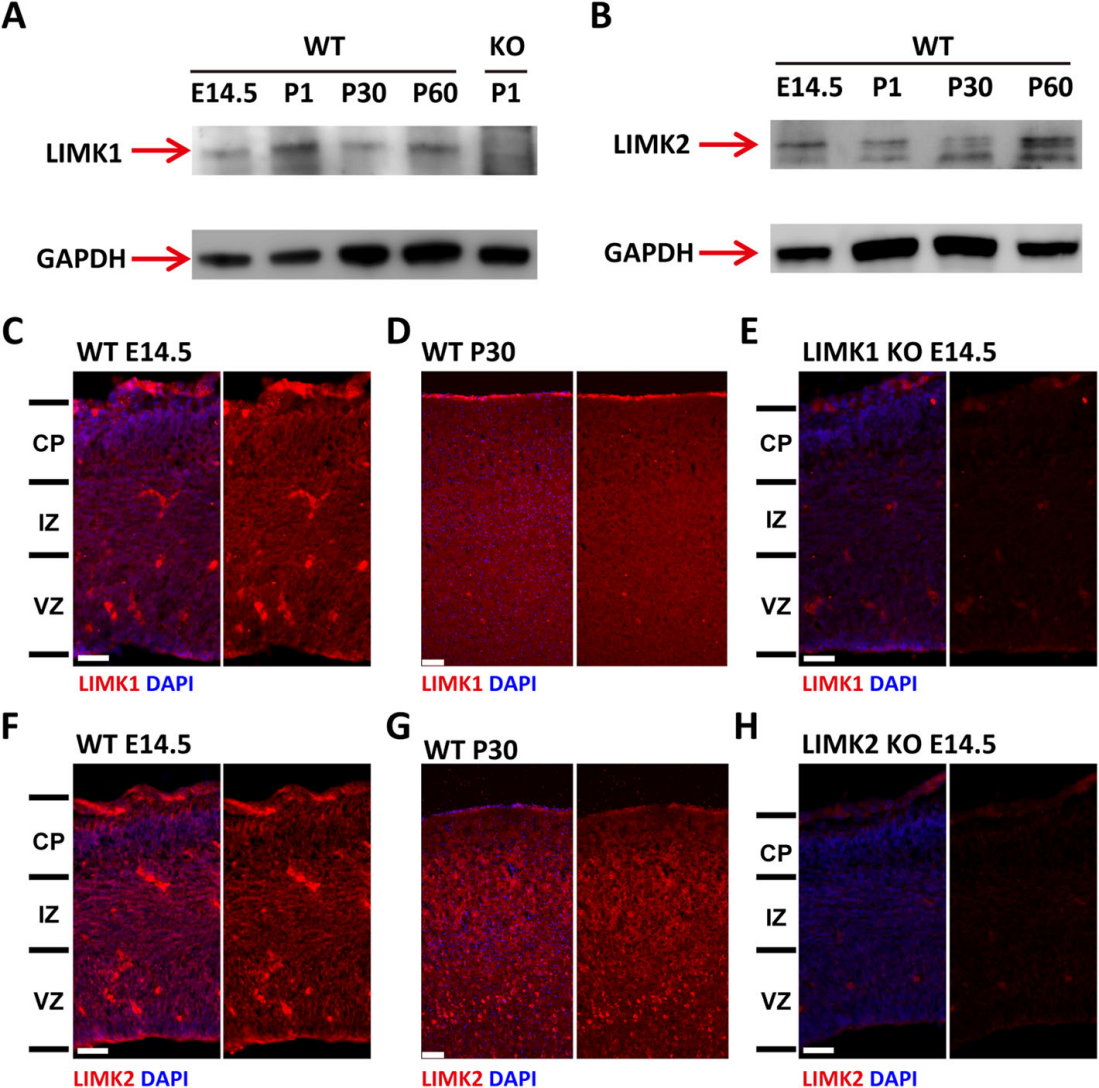
Expression of LIMK1 and LIMK2 in developing mouse neocortex. **a**, **b** Western blots showing LIMK1 (**a**) and LIMK2 (**b**) expression in mouse neocortex at various developmental stages. (**c**, **d**, **e**) Coronal brain sections co-stained with anti-LIMK1 and DAPI showing the expression of LIMK1 in WT E14.5 (**c**) and WT P30 (**d**), but not in LIMK1 KO E14.5 (**e**). (**f**, **g**, **h**) Coronal brain sections co-stained with anti-LIMK2 and DAPI on coronal brain sections of WT E14.5 (**f**) and WT P30 (**g**), but not in LIMK2 KO E14.5 (**h**). Note some remaining staining signals in LIMK1 and LIMK2 KO sections, suggesting that the antibodies might be non-specific. CP, cortical plate; IZ, intermediate zone; VZ, ventricular zone. Scale bars, 50 μm

**Fig. 2:**
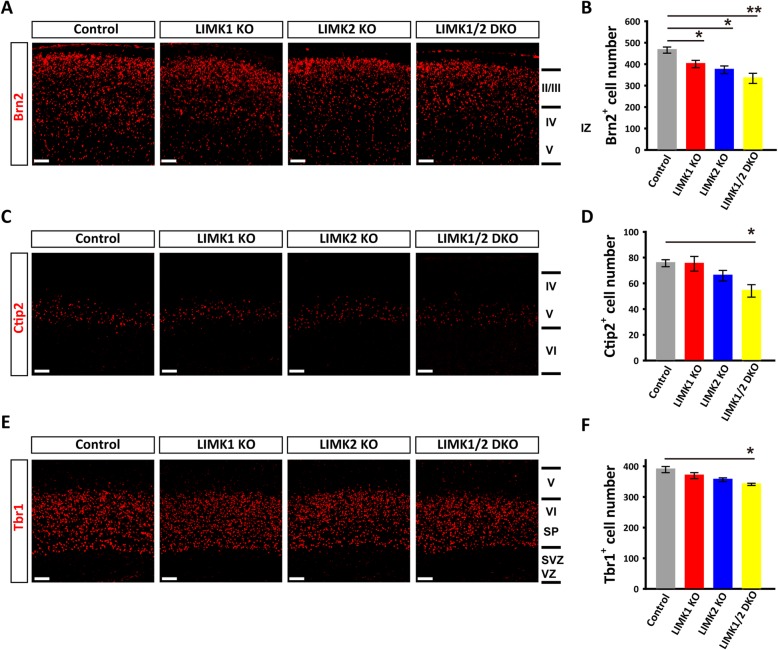
Altered layer-specific markers in LIMK1 KO and LIMK2 KO cortex. **a**, **b** Sample coronal brain sections of postnatal day 0 (P0) stained for the late-born neuronal marker Brn2 (layer II and III) and summary graph showing reduced Brn2^+^ neurons in LIMK2 KO and LIMK1/2 DKO mice. **c** and **d** Sample coronal brain sections stained for the layer V neuronal marker Ctip2 and summary graph showing reduced Ctip2^+^ neurons in LIMK1/2 DKO mice. **e** and **f** Sample coronal brain sections stained for the layer VI neuronal marker Tbr1 and summary graph showing a trend of reduction in Tbr1^+^ neurons in LIMK2 KO and LIMK1/2 DKO mice. WT *n* = 7, LIMK1 KO *n* = 6, LIMK2 KO *n* = 6, LIMK1/2 DKO *n* = 5. Data are presented as mean ± SEM. *p < 0.05, **p < 0.01, ***p < 0.001, one-way ANOVA, Dunnett’s test. Scale bars, 50 μm

Although LIMK1 and LIMK2 show significant structural similarities, their protein expression, subcellular localization and functions are considerably different [29]. Therefore, it is possible that LIMK1 and LIMK2 may play different roles in cortical development. To investigate this possibility, we first detected the expression of LIMK1 and LIMK2 in mouse brain from embryonic day to adult. The result of western blot and immunofluorescent staining confirmed that both LIMK1 and LIMK2 are expressed in developing embryonic brain (Fig. 1). Consistent with previous reports, LIMK1 was mainly localized in the neurites while isoforms of LIMK2 differentially expressed at diverse developmental stage and show different subcellular localization [17, 30, 31]. We then analyzed the overall cellular organization of the cortex of LIMK1 KO, LIMK2 KO, LIMK1/2 DKO and their WT littermates using layer-specific markers. The P0 pups were used since the production of cortical neuron have finished by this time. As shown in Fig. 2, the distribution of the layer-specific markers was similar in all genotypes, indicating that the overall laminar organization of the cortex was not grossly altered in these mice (Fig. 2). However, LIMK KO mice showed reduced late born neurons (Brn2^+^) compared to WT littermates (Fig. 2a and b). In addition, the Ctip2^+^ and Tbr1^+^ neurons in the middle layers were also reduced in LIMK1/2 DKO mice (Fig. 2c-f). These data indicate that LIMKs are important for normal cortical development.

### Altered cortical distribution of BrdU^+^ neurons in LIMK KO mice

**Fig. 3:**
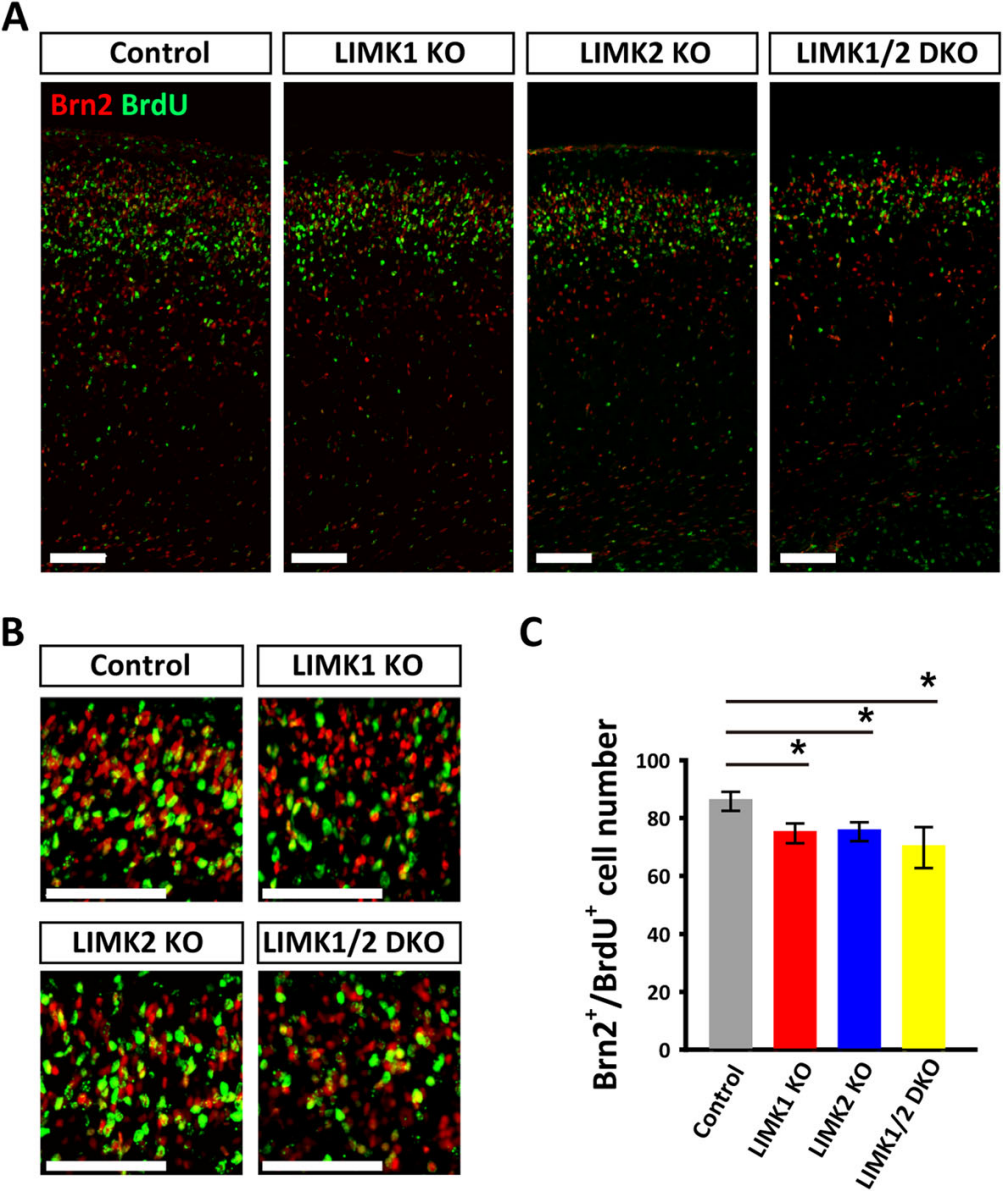
Reduced pyramidal neurons in the upper cortical layer of LIMK KO mice. **a** and **b** Sample coronal brain sections stained for the late-born neuronal marker Brn2 (red) and BrdU (green) injected at E14.5, showing co-location of Brn2^+^ and BrdU^+^ neurons (yellow) in the upper layer of P0 cortex. **c** Summary graph showing reduced Brn2^+^/BrdU^+^ neurons in LIMK1 KO, LIMK2 KO and LIMK1/2 DKO mice. WT *n* = 6, LIMK1 KO *n* = 5, LIMK2 KO *n* = 5, LIMK1/2 DKO *n* = 3. Data are presented as mean ± SEM. *p < 0.05, one-way ANOVA, Dunnett’s test. Scale bars, 50 μm

**Fig. 4:**
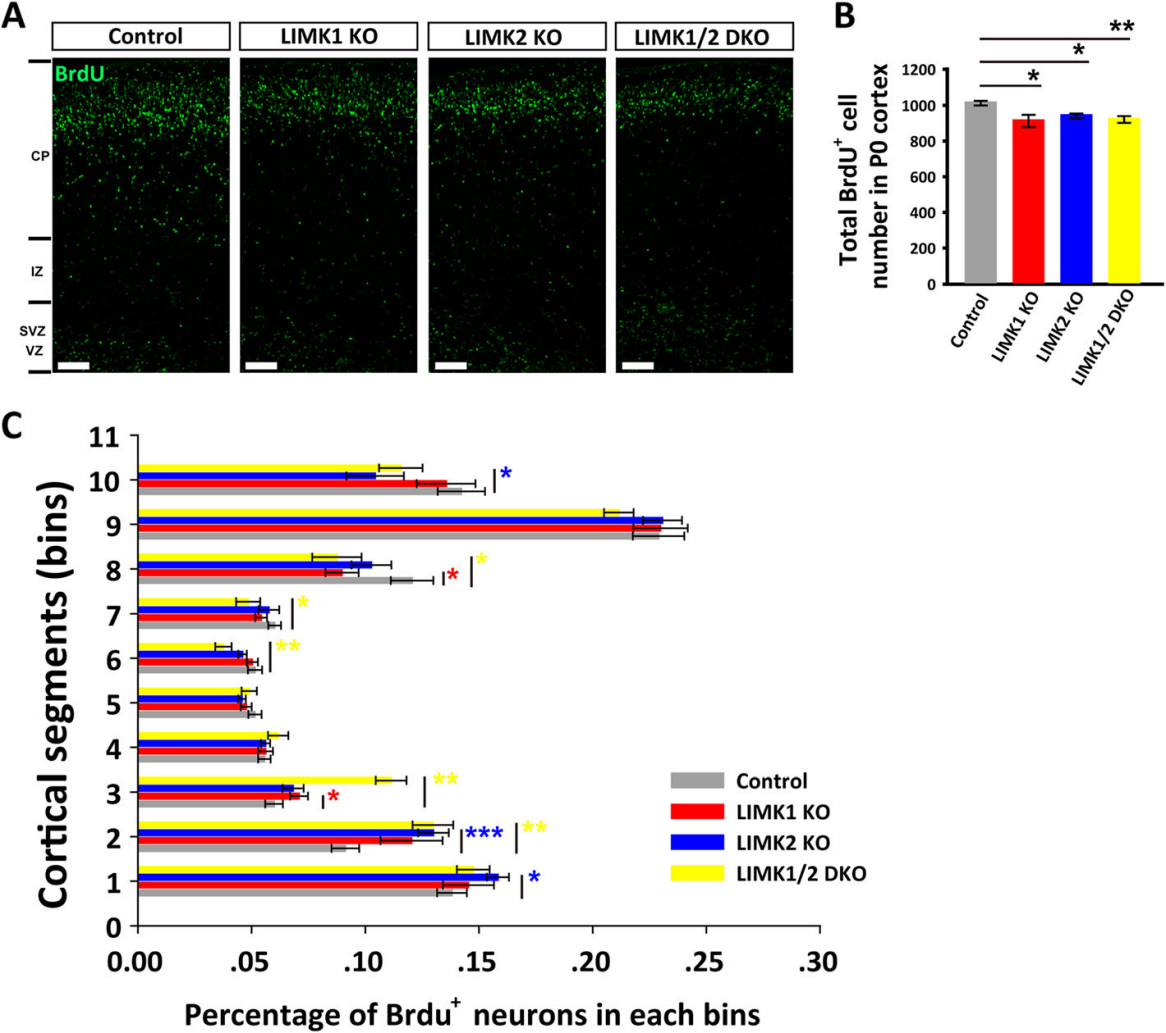
Impaired neuronal migration in in LIMK KO mice. **a** Pregnant mice were labeled with BrdU at E14.5 and postnatal day 0 (P0) pups were stained for BrdU showing reduced BrdU^+^ cells in LIMK1 KO, LIMK2 KO and LIMK1/2 DKO mice. **b** Summary graph showing reduced total BrdU^+^ cells in LIMK1 KO, LIMK2 KO and LIMK1/2 DKO mice. **c** Distribution of BrdU^+^ cells across the cortex from (**a**), 1–10 represent the spatial order from VZ/SVZ to CP. WT control, *n* = 6; LIMK1 KO, *n* = 5; LIMK2 KO, *n* = 5; LIMK1/2 DKO, *n* = 3. Data are presented as mean ± SEM. Statistical significance was determined by one-way ANOVA, Dunnett’s test. *p < 0.05, **p < 0.01. Scale bars, 50 μm

Having known that the late born pyramidal neurons were decreased in LIMK1 KO, LIMK2 KO and LIMK1/2 DKO mice, we then asked whether the loss of late born pyramidal neurons was a result of impaired neural migration and/or proliferation. We injected pregnant mice with BrdU at E14.5, a time window when the late born pyramidal neurons (laye II and layer III) are generated and migrating, to label the proliferating neural progenitors cells and their progeny. We first analyzed the number of BrdU labeled Brn2^+^ neurons at P0. Compared to control mice, the number of BrdU^+^/Brn2^+^ neurons was decreased in LIMK1 KO, LIMK2KO and LIMK1/2 DKO mice (Fig. 3). We also analyzed the distribution of all BrdU^+^ neurons across the cortical plate. The neocortex was divided into equal 10 bins and the percentage of BrdU^+^ neurons in each bin was quantified. As shown in Fig. 4, the total number of BrdU^+^ neurons was significantly lower in LIMK1 KO, LIMK2 KO and LIMK1/2 DKO mice compared to WT control (Fig. 4a, b). It is important to note that although the total number of BrdU^+^ neurons was reduced, their fractions in the VZ/SVZ area were significant greater compared to WT control (Fig. 4a and c). These results suggest that LIMK1/2 are involved in both neural proliferation and neuronal migration.

### Impaired neural progenitor cell proliferation in LIMK KO mice

**Fig. 5:**
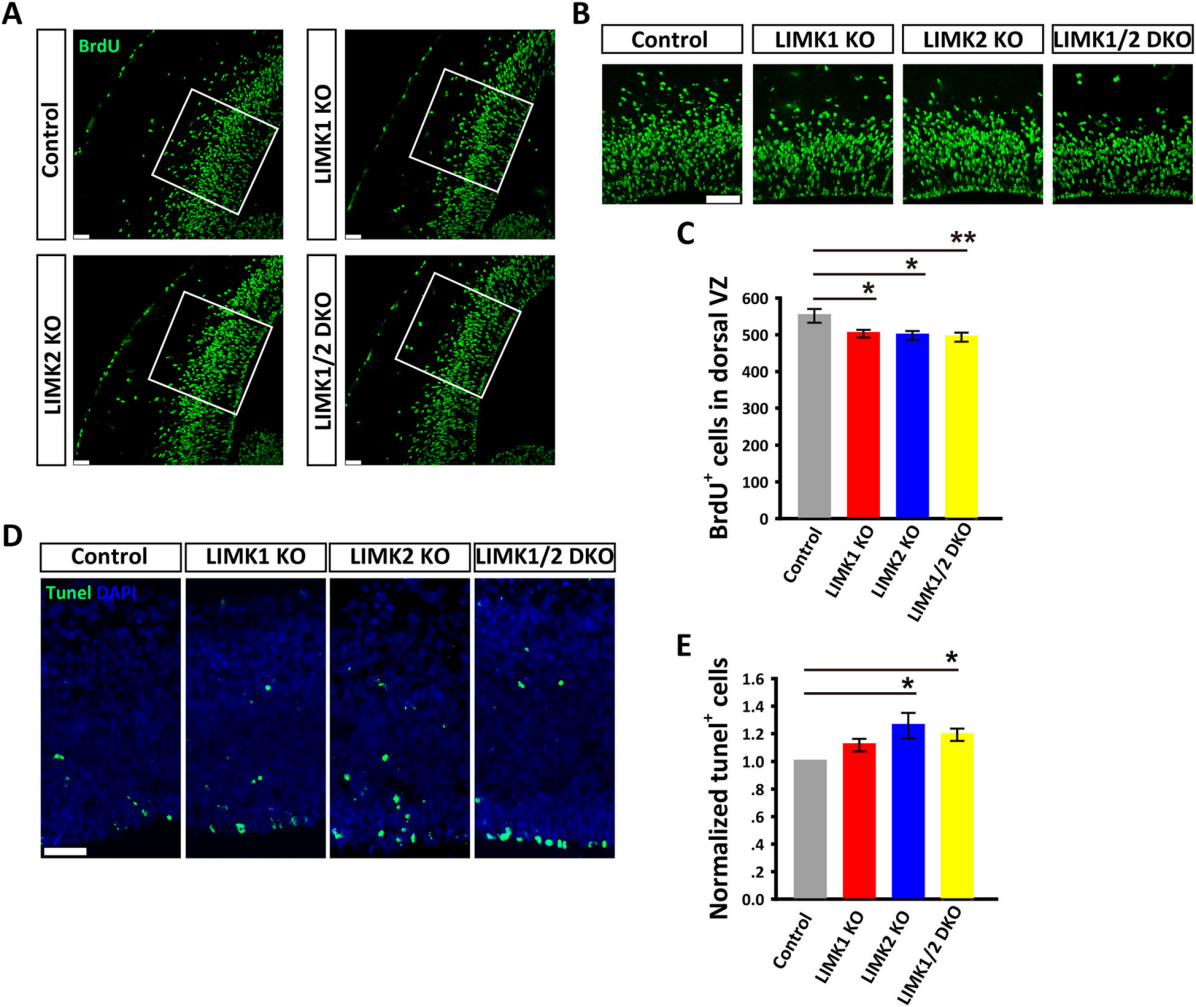
Reduced cell proliferation in the dorsal telencephalon of LIMK KO mice. **a** Pregnant mice were injected with BrdU at E14.5 and 2 h later the embryos were dissected and stained for BrdU. **b** Magnified views of the boxed regions in (**a**). **c** Summary graph of (**a**) showing a significant reduction of BrdU^+^ cells in the dorsal VZ in various LIMK KO embryos. **d** Coronal brain sections of E14.5 embryos stained for TUNEL (green) and DAPI (blue). **e** Summary graph showing significantly increased numbers of TUNEL positive cells in LIMK2 KO and LIMK1/2 DKO mice. Control, *n* = 4; LIMK1 KO, *n* = 5; LIMK2 KO, *n* = 3; LIMK1/2 DKO, *n* = 3. Data are presented as mean ± SEM. Statistical significance was determined by one-way ANOVA, Dunnett’s test. *p < 0.05, **p < 0.01, ***p < 0.001. Scale bars, 50 μm

To further investigate the nature of the reduced BrdU^+^ neurons in LIMK KO mice, we injected the pregnant mice at E14.5 with a high concentration of BrdU (200 mg/kg) for a brief period of time (i.e., 2 h) and the brains of the embryos were then immediately fixed and sectioned for BrdU staining. This allowed an estimate of the rate of neural progenitor cell proliferation at this particular time. As shown in Fig. 5a-c, the number of BrdU^+^ proliferating progenitors was significantly reduced in LIMK1 KO, LIMK2 KO and LIMK1/2 DKO mice compared to WT mice, suggesting that both LIMK1 and LIMK2 are important for cell proliferation. To determine whether there were alterations in cell death, we performed the TUNEL staining for apoptotic cells. As shown in Fig. 5d and e, increased staining was observed in the dorsal telencephalon of LIMK2 KO and LIMK1/2 DKO mice, but not in LIMK1 KO mice. These results suggest that although both LIMK1 and LIMK2 are involved in neural proliferation, only LIMK2 may play a role in embryonic cell apoptosis.

### Reduced neural progenitors in LIMK KO mice

**Fig. 6:**
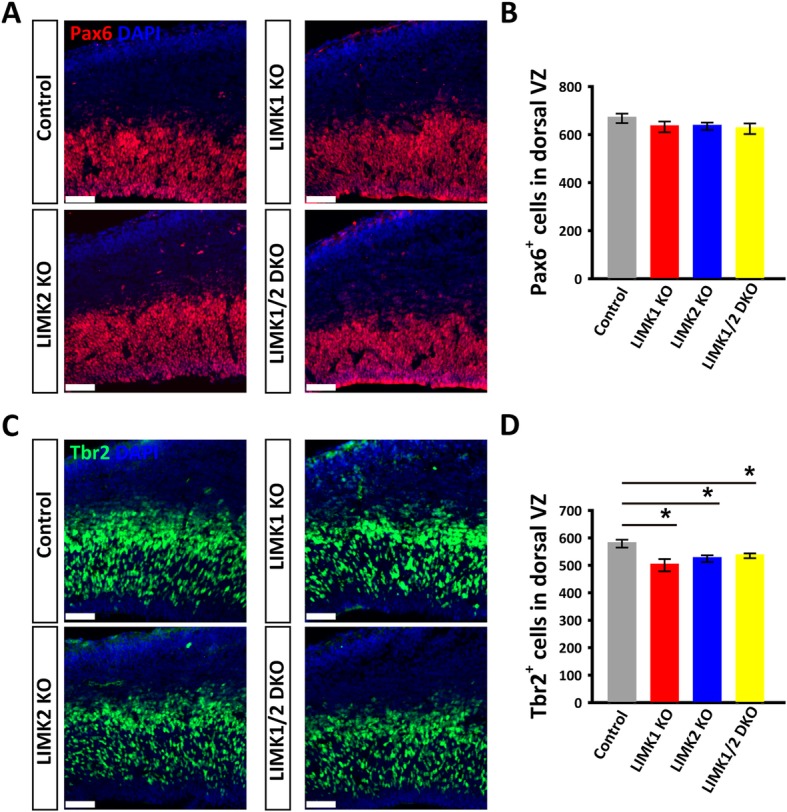
Coronal brain sections of WT control and LIMK KO mice E14.5 embryos were stained for Pax6 or Tbr2. **a** Pax6 (red) and DAPI (blue) staining of WT control and various LIMK KO mice. **b** Summary graph of Pax6 positive cells showing no differences between control and various LIMK KO mice. **c** Tbr2 (green) and DAPI (blue) staining of WT control and various LIMK KO mice. **d** Summary graph showing significantly decreased numbers of Tbr2 positive cells in LIMK1 KO, LIMK2 KO and DKO mice compared to their control littermates. Control, *n* = 6; LIMK1 KO, *n* = 3; LIMK2 KO, *n* = 5; LIMK1/2 DKO, *n* = 3. Data are presented as mean ± SEM. Statistical significance was determined by one-way ANOVA, Dunnett’s test. *p < 0.05, **p < 0.01. Scale bars, 50 μm

To further determine the identity of the BrdU^+^ cells in VZ/SVZ area, we performed immunostaining for Pax6 (a marker for self-renewal neural progenitor cells) and Tbr2, (a marker for intermediate progenitors) on the E14.5 brain sections. As shown in Fig. 6, while the number of Pax6^+^ progenitors showed no changes in any of the LIMK KO mice, the Tbr2^+^ progenitors were significantly reduced in LIMK1 KO, LIMK2 KO and LIMK1/2 DKO mice compared to WT control. These results suggest that LIMKs regulate neural progenitor cells primarily through affecting intermediate progenitors.

### LIMKs regulate cell cycle progression

**Fig. 7:**
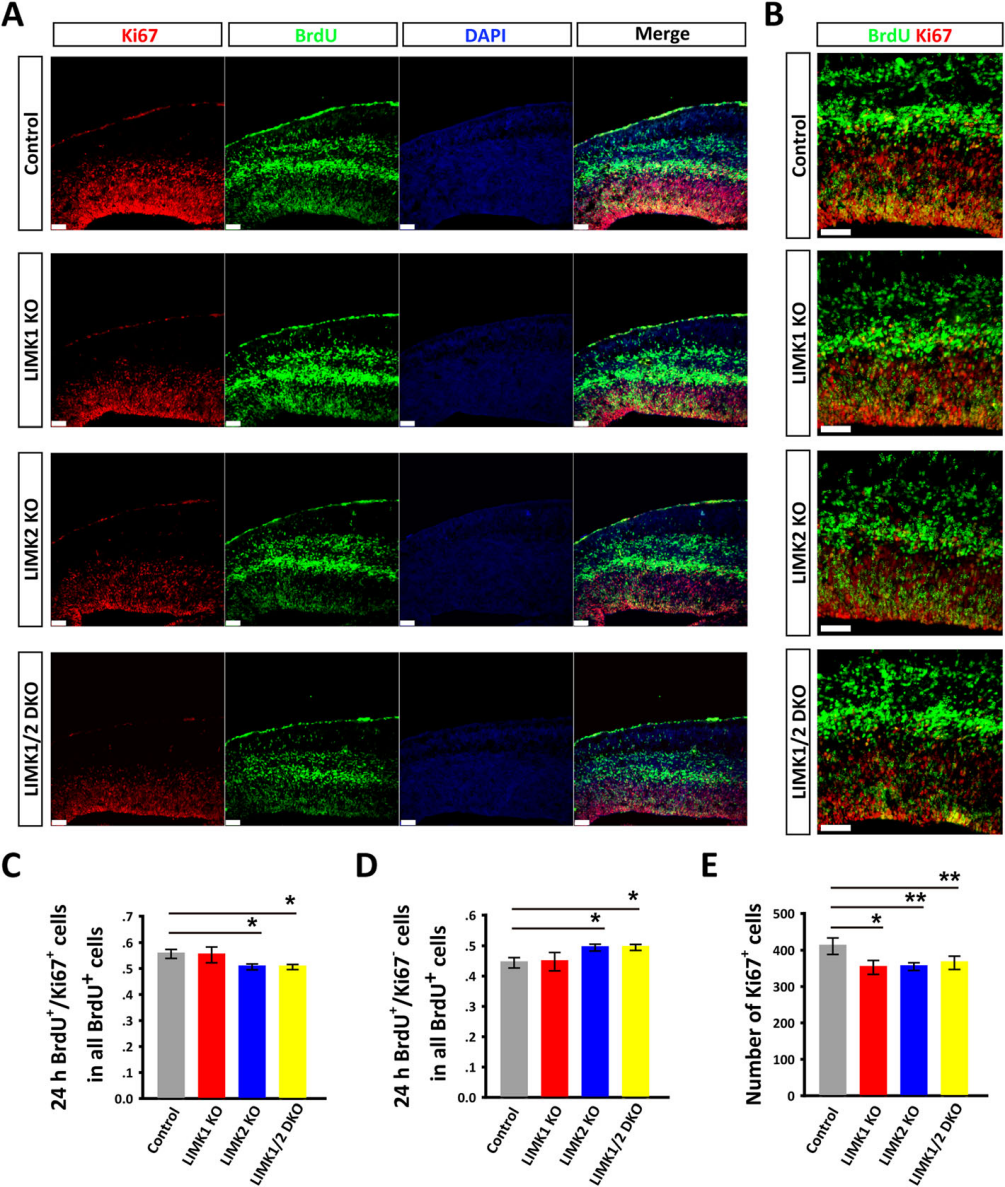
Altered cell cycle state in the telencephalon of LIMK KO mice. **a** Pregnant mice were injected with BrdU at E13.5 for 24 h and the embryos of the control and various LIMK KO were then dissected at E14.5 and processed for immunostaining for BrdU (green), Ki67 (red) and DAPI (blue). **b** Magnified views of the co-localization of BrdU^+^/Ki67^+^ cells. **c**-**e** Summary graphs of the percentages of BrdU^+^/Ki67^+^(**c**) and BrdU^+^/Ki67^−^ (**d**) cells relative to the total BrdU^+^ cells showing a significantly decreased number of cells staying in the active phases of the cell cycle, as indicated by reduced BrdU^+^/Ki67^+^ cells and increased BrdU^+^/Ki67^−^ cells, in LIMK KO mice compared to the control littermates. Summary graph showing that the number of total Ki67^+^ cells (**e**) was also decreased in the dorsal telencephalon in the LIMK KO mice compared to the control littermates. Control, *n* = 6; LIMK1 KO, *n* = 3; LIMK2 KO, *n* = 5; LIMK1/2 DKO, *n* = 3. Data are presented as mean ± SEM. Statistical significance was determined by one-way ANOVA, Dunnett’s test. *p < 0.05, **p < 0.01. Scale bars, 50 μm

**Fig. 8:**
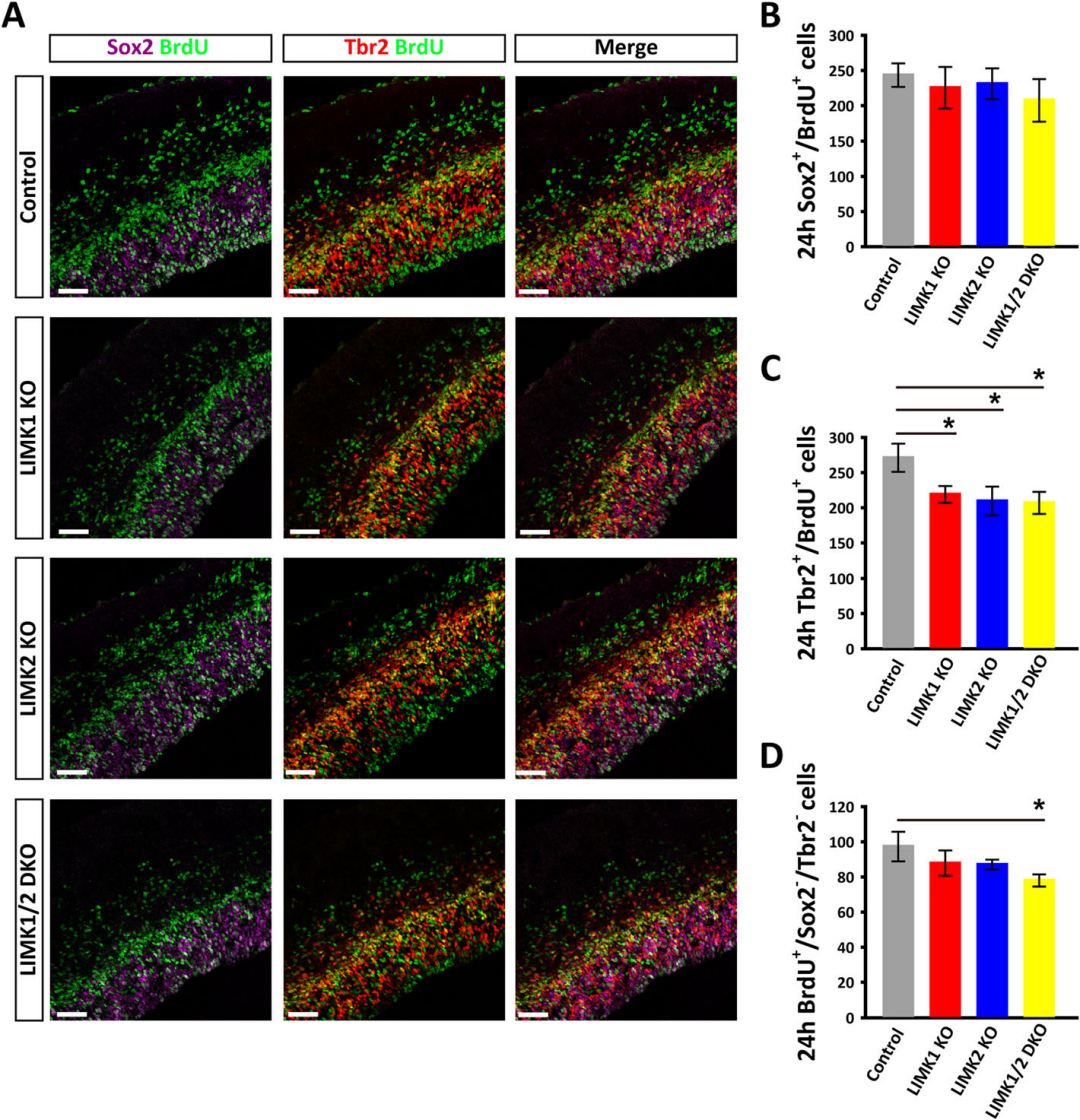
Reduced proliferation of intermediate neural progenitors in LIMK KO mice. **a** Pregnant mice were injected with BrdU at E13.5 for 24 h and the embryos of the control and various LIMK KO were then dissected at E14.5 and processed for immunostaining for Sox2 (purple), Tbr2 (red) and BrdU (green). **b**-**d** Summary graphs of the number of Sox2^+^/BrdU^+^(**b**), Tbr2^+^/BrdU^+^(**c**) and BrdU^+^/Sox2^−^/Tbr2^−^ cells (**d**).Although the numbers of newborn radial glial cell (Sox2^+^/BrdU^+^), intermediate progenitor (Tbr2^+^/BrdU^+^) and neurons (BrdU^+^/Sox2^−^/Tbr2^−^ cells) all showed a trend of decrease in LIMK KO mice compared to the control littermates, only the reduction of intermediate progenitors (Tbr2^+^/BrdU^+^) showed a significant difference. Control, *n* = 6; LIMK1 KO, *n* = 3; LIMK2 KO, *n* = 5; LIMK1/2 DKO, *n* = 3. Data are presented as mean ± SEM. Statistical significance was determined by one-way ANOVA, Dunnett’s test. *p < 0.05, **p < 0.01. Scale bars, 50 μm

To investigate how LIMKs regulate neural proliferation, we examined the cell cycle state (active or inactive) of neural progenitor cells in LIMK KO mice. The pregnant mice were injected with BrdU (50 mg/kg) at E13.5, and 24 h later the brain sections were co-stained for Ki67 (a marker for the active phases of the cell cycle) and BrdU. The data showed that the number of active proliferating cells (BrdU^+^ and Ki67^+^) was significantly decreased in LIMK2 KO and LIMK1/2 DKO mice (Fig. 7a-c). Accordingly, the number of cells that exited from the cell cycle (BrdU^+^ and Ki67^−^) was increased in LIMK2 KO and LIMK1/2 DKO mice (Fig. 7a-d). In addition, the total number of Ki67^+^ cells was decreased in all three LIMK KO mice (Fig. 7e). To further investigate whether proliferation of radial glia cells and intermediate progenitor cells were affected differently, we co-stained Sox2 and Tbr2 in BrdU incorporated sections. The result showed that only the number of intermediate progenitor cells (Tbr2^+^/BrdU^+^) was significantly decreased (Fig. 8). These results suggested that LIMKs are particularly important in maintaining the proliferating capacity of the intermediate progenitor cells.

## Discussion

It is well established that LIMK 1 and 2 are key players in the regulation of the actin cytoskeleton through phosphorylation and inactivation of cofilin [18–20, 27]. Given the critical importance of the actin cytoskeleton in various cellular functions, particularly neuronal morphology and motility, it is not surprised that genetic ablation of the LIMK1/2 caused profound impairments in neuronal structure and function, including axonal and spine morphology, synaptic plasticity and memory in adult mice [22–26]. However, whether LIMK1/2 are also involved in brain development remains unknown. In this study, we investigated the role of LIMK1/2 in mouse neocortical development by examining neural progenitor cell proliferation, neuronal migration and neocortical layer organization in developing LIMK1 KO, LIMK2 KO and LIMK1/2 DKO mice. We showed that these KO mice exhibit significant deficits in both neural proliferation and migration, suggesting that LIMK-mediated actin is a critical player in these processes. In addition, we show that LIMK1 KO and LIMK2 KO mice are differentially altered in cell proliferation and apoptosis, suggesting that LIMK1 and LIMK2 may play distinct roles in some aspects of brain development. Our results provide strong in vivo evidence for the critical involvement of LIMK signaling and actin in embryonic brain development.

The initiation of neuronal migration is activated by the of membrane receptors through local environmental cues followed by the polarization of the actin cytoskeleton and the extension of the leading process of the cell [32, 33]. As key regulators of actin dynamics, LIMKs have been proved to play regulatory role in cell motility, particularly in tumor cell invasion, including human breast cancer cell lines and Jurkat T cells [34–36]. In contrast, their function in neuronal migration remains unknown. In this study, we demonstrated that the upper layer pyramidal neurons are reduced in LIMK1/2 KO mice compare to the WT littermates. These results are similar to those observed in PAK1 KO mice [12], suggesting that LIMKs are downstream targets of PAK1 in the regulation of cortical neuronal migration.

We also find that LIMK KO mice are impaired in the proliferation of neural progenitor cells, which is similar to the deficits reported in the PAK1 KO mice [12]. In LIMK KO mice, the number of progenitor cells at E14.5, especially the intermediate progenitors, is significantly less than that in control mice. Previous studies in cultured cell lines have shown that LIMKs participate in several steps of cell cycle progression [37–39]. LIMK1 and LIMK2 have distinct subcellular localization and play different roles during cell division [40, 41]. Inhibition of LIMK1 activity during mitosis causes activation of cofilin and lead to delayed transition from metaphase to anaphase and irregular spindle positioning, while LIMK2 is important for normal mitotic spindle formation [38, 39, 42]. During cortical development, neural progenitor fata decision and neurogenesis is closely associated with asymmetric and symmetric cell division, when the accurate spindle positioning and orientation is essential for cells to determine the fates of daughter cells after mitosis [43, 44]. In addition, the length of cell cycle is one of the main elements that affect the final output of total neurons [45–47]. The differential role played by LIMK1 and LIMK2 in spindle positioning and formation, respectively, may have different effects on cell division and cell death. This may be responsible for specific deficits in LIMK2 KO mice where we observed increased apoptotic cell death, which is not detected in LIMK1 or PAK1 KO mice. Our results are consistent with a previous study showing that LIMK2 knockdown in neuroblastoma cells increased the sensitivity to microtubule-targeted drugs and caused enhanced apoptosis [38]. Our results that KO mice for LIMK1, LIMK2 or PAK1 have similarities and difference in changes in embryonic neuronal proliferation and cell cycle progression provide in vivo evidence that they have both shared and distinct functions in these processes.

In conclusion, we show here that LIMK1 KO and LIMK2 KO mice are impaired in neuronal migration and progenitor cell proliferation with some changes unique to one or the other KO mice. These impairments are similar to those found in PAK1 KO mice and suggest that LIMK-dependent actin reorganization plays a key role in mediating the effects of PAK1 in the regulation of the cortical development.

## Methods

### Animals

The generation and initial characterization of LIMK1 and LIMK2 KO mice were described previously [23, 48]. The LIMK-1/2 DKO mice were obtained from LIMK-1 +/− LIMK-2 +/− interbreeding. To minimize the effect of genetic background, all the pups and embryos used in this study were LIMK1/2 KOs and their WT or heterozygotes littermates derived from the heterozygous breeders. All the mice were housed under a standard 12-h light/12-h dark cycle condition at the Experimental Animal Center at Southeast University, China. All animal experiment procedures were approved by Southeast University (Nanjing, China) and the Hospital for Sick Children (Toronto, Canada) Animal Care and Use Committee.

### Tissue processing and immunohistochemistry

Procedures for brain processing and immunohistochemistry were described previously [12]. Briefly, for embryonic 14.5 (E14.5) samples, the pregnant mice were anesthetized by intraperitoneal injection with 7% chloral hydrate (10 μl/g). The fetal brains were dissected and fixed with 4% paraformaldehyde (PFA) for 6 h at 4 °C, and PFA was replaced with 30% sucrose dissolved in PBS at 4 °C until the brains were saturated. For postnatal samples, the neonatal mice were anaesthetized by hypothermia, then subjected to cardiac perfusion with 0.1 M phosphate buffered saline (PBS), followed by 4% PFA. The brains were dissected and fixed with 4% PFA overnight at 4 °C. The PFA was replaced with 30% sucrose dissolved in PBS at 4 °C until the brains were saturated. The saturated brains were embedded in Tissue-Tek® O.C.T. Compound and frozen by liquid nitrogen. The samples were cut into coronal sections of 10 μm for prenatal day brains and 16 μm for postnatal day brains using a Leica CM1950 cryostat. Brain sections were washed in PBS for 10 min, permeabilized with 0.1% Triton X-100 in PBS, blocked with 0.1% Triton X-100, 10% fetal bovine serum in PBS for 2 h at room temperature, then incubated with primary antibodies overnight at 4 °C. Subsequently, sections were washed in 0.1% PBT and incubated in appropriate secondary antibodies for 2 h at 37 °C.

### Antibodies and reagents

Primary antibodies used in this study include: rabbit anti-Brn2 (Santa Cruz, 1:50), rabbit anti-Ctip2 (Abcam, 1:1000), rabbit anti-Tbr1 (Proteintech Group, 1:500), rat anti-BrdU (Abcam, 1:1000), rabbit anti-Ki67 (Abcam, 1:500), mouse anti-Pax6 (Cell Signaling Technology, 1:200), rabbit anti-Pax6 (Proteintech Group, 1:1000). Secondary antibodies used include: Alexa Fluor 555 donkey anti-rabbit IgG (Invitrogen, 1:300), Alexa Fluor 488 donkey anti-rabbit IgG (Invitrogen, 1:300), Alexa Fluor 488 goat anti-mouse IgG (Proteintech Group, 1:300), Alexa Fluor 488 goat anti-rat IgG (Jackson Immuno Research, 1:300). Cell apoptosis was detected by TUNEL FITC Apoptosis Detection Kit (Vazyme, A111). Nuclei were counterstained with 4, 6-diamidino-2-phenylindole (DAPI; Cayman Chemical).

### Microscope setups and image collection

Immunostaining images were collected at room temperature using confocal laser microscopes (LSM700, Carl Zeiss) or a light microscope (CTR 5000; Leica) at 2,048 × 2,048 pixels. The images were then analyzed with Zeiss Aim Image Browser software or Image J software. In postnatal experiments, greater than 3 brains of each genotype from at least 5 litters were used for analysis. At least 3 discontinuous coronal sections from somatosensory cortex from each brain were counted. For the migration study, BrdU^+^ cells were measured in vertical strips with a 350 μm width and the cortical slice images were equally divided into ten bins across the six-layered cortex. The regions of cortex were identified by layer specific markers and the DAPI staining. In embryonic studies, greater than 3 brains of each genotype from at least 6 litters were used for immunostaining experiments. No less than 4 discontinuous coronal sections of the medial cortex from each brain were analyzed for cell counts. For each section, an area of 300–400 μm wide with a length spanning either VZ/SVZ or the entire middle regions of the E14.5 telencephalon was analyzed. The labeled cells were manually counted use the Cell Counter plugin of Image J software.

### Statistical analysis

One-way ANOVA, Dunnett’s test was used to evaluate the data statistically, and when *P* < 0.05, the differences were considered significant. And all data were presented as means ± standard errors of the means (SEM).

## Data Availability

The data used in our study are available from the authors on reasonable request.

## Abbreviations

BrdU: Bromodeoxyuridine
Brn2: Pou3f2 (POU domain, class 3, transcription factor 2)
Ctip2: Bcl11b (B cell leukemia/lymphoma 11 B)
Pax6: Paired box 6
Tbr1: T-box, brain 1
TUNEL: Terminal-deoxynucleoitidyl Transferase Mediated Nick End Labeling
